# Cuticular Hydrocarbon Differentiation Between Body Parts of *Schistocerca gregaria* Locusts

**DOI:** 10.1007/s10886-025-01687-y

**Published:** 2026-02-12

**Authors:** Selina Huthmacher, Florian Menzel

**Affiliations:** Institute of Organismic and Molecular Evolution, Johannes Gutenberg-Universität Mainz, Mainz, Germany

**Keywords:** Cuticular hydrocarbons, Locust, Chemical communication, Surface lipids, Multifunctionality

## Abstract

Insects cover their bodies with a layer of cuticular hydrocarbons (CHCs) that reduce water loss and serve as communication signals and potentially as lubricants. CHC composition is highly diverse, varying among individuals and species. However, CHC variation has been rarely studied between different body parts of the same individual. Whether intra-individual CHC variation across the insect body arises from differences in diffusion rates between different CHCs or from distinct functional roles of the CHC layer across body regions remains unknown. Here, we analyzed the CHC composition of antennae, head, thorax, abdomen, front legs, hind femur and tibia, as well as whole-body extracts of the desert locust *Schistocerca gregaria* subadults using gas chromatography-mass spectrometry (GC–MS). While most body parts were relatively similar in CHC composition, head and antennae were distinct from the others. Both had shorter *n-*alkanes and more branched alkanes than the other body parts, which should lower the CHCs’ melting temperatures and hence render the CHC layer more fluid. On the antennae, this likely reflects their role as sensory organs in chemical communication, as more fluid CHCs have been suggested to be more easily perceived. We additionally examined the femoro-tibial joint of the hind leg using solid-phase microextraction (SPME). The joint contained fewer and shorter *n*-alkanes than the femur, indicating a relative enrichment of more fluid CHCs, which may facilitate lubrication. Together, these results show that CHC composition in *S. gregaria* varies systematically across body regions. This likely reflects that CHCs are optimised for distinct functions in desiccation resistance, chemical communication, and mechanical lubrication, depending on the body part.

**Supplementary Information:**

The online version contains supplementary material available at 10.1007/s10886-025-01687-y.

## Introduction

Protection against desiccation is critical for all terrestrial species. Insects are particularly vulnerable to water loss because of their high surface-to-volume ratio, with up to 80% of total water loss occurring through body surfaces (Blomquist and Bagnères 2010; Gibbs 1998; Gibbs and Rajpurohit 2010).

To prevent desiccation, insects cover their cuticle with a layer of cuticular hydrocarbons (CHCs) (Edney 1977; Hadley 1994). These CHCs establish a passive barrier that mitigates water loss by reducing the outward diffusion of water (Chown et al. 2011; Edney 1977; Krupp et al. 2020). CHCs additionally function as vital signals for chemical communication, both in social insects (Leonhardt et al. 2016) and in solitary species (e.g. in a mating context Steiger et al. 2013; Thomas and Simmons 2008, 2011; Weddle et al. 2013). Furthermore, CHCs have been suggested to act as lubricants (Cooper et al. 2009), and improve foot adhesion to surfaces (Drechsler and Federle 2006). The main CHC classes are *n*-alkanes, methyl branched alkanes and unsaturated CHCs (Blomquist and Bagnères 2010), which differ in the number and position of methyl groups and/or double bonds. Pure *n*-alkanes exhibit the tightest aggregation due to intermolecular van der Waals interactions and are typically solid at room temperature (Maroncelli et al. 1982). Molecular aggregation is stronger in hydrocarbons with longer chain lengths (Gibbs & Rajpurohit 2010). Double bonds (alkenes and alkadienes) and methyl branches (methyl alkanes, or, rarely, methyl alkenes) disrupt molecular aggregation of the CHCs, leading to lower melting points, and, at a given temperature below the melting point, to lower viscosities. Methyl group position also influences the melting point and viscosity, such that internally branched methyl alkanes are more fluid than more terminally branched ones (Gibbs and Pomonis 1995). Previous studies showed that at ambient temperatures, insect CHCs occur as a mixture of solid (gel-like) and liquid but highly viscous phases (Menzel et al. 2019; Huthmacher et al. 2025). Viscosity always decreases with increasing temperature but is only defined for liquid materials. For the sake of brevity, CHCs with lower melting points and lower viscosity at a given temperature will henceforth be referred to as ‘more fluid’. The CHC layer of an individual insect can comprise up to 100 distinct compounds, each consisting of chains ranging from 20 to 40 carbon atoms (Blomquist and Bagnères 2010; Golian et al. 2022). This composition (the CHC profile) is highly diverse across species, even between sister taxa (Foley et al. 2007; Hartke et al. 2019; Pokorny et al. 2014; Sprenger and Menzel 2020). The diversity in CHC composition may be partly driven by sexual selection (Veltsos et al. 2012), as well as adaptation to different ecological niches (Frentiu and Chenoweth 2010; Menzel et al. 2017; Wang et al. 2022).

However, differences in CHC composition are not only observed between taxa, but also within a single individual across different body parts. It was shown that melting temperatures of the CHC layer vary between body parts in *Drosophila melanogaster* fruit flies and in *Melanoplus* grasshoppers (Gibbs and Crowe 1991; Wang et al. 2016b) suggesting a different CHC composition between body parts. In various ant species, thorax, gaster, and legs often differed in quantitative CHC composition. Compared to thorax and legs, the gaster was often enriched in more fluid compounds like alkenes and alkadienes. Most notably, the postpharyngeal gland differed from most other body parts, showing less *n-*alkanes, but more alkenes, and more internally branched monomethyl alkanes, dimethyl and trimethyl alkanes (Sprenger et al. 2021). Furthermore, antennal CHC profiles seem to differ from other body parts in *Iridomyrmex* ants (Wang et al. 2016a). 

These body part-specific CHC variations may be explained by several possible mechanisms. One possibility is the selective transport of some CHCs to particular regions of the cuticle, a process that has been observed in sex pheromones of moths, where long-chain aliphatic compounds are selectively transported to specific glands (Blomquist and Bagnères 2010; Jurenka et al. 2003; Schal et al. 1998). In this case, CHC differentiation should be linked to their functions. In chemical communication, antennae are crucial not only as sensory organs, but also as potential sources of chemical signals, with both social and solitary insects using them to detect conspecific cues through olfactory sensilla (Ozaki and Wada-Katsumata 2010; Ryan and Sakaluk 2009). Chemical communication may be less important for the remaining body parts, such that here, CHC profiles may rather be optimised for desiccation resistance. Accordingly, body parts with a higher surface-to-volume ratio are expected to be covered by more solid CHCs to minimize water loss. Furthermore, CHCs also act as joint lubricants (Cooper et al. 2009), such that joints should be enriched in lower-melting CHCs compared to surrounding body parts.

All CHCs are produced in oenocytes in the fat body, which is most abundant in the abdomen, and then transported through the hemolymph onto the cuticle by lipophorins (Haruhito & Haruo 1982; Fan et al. 2004). Thus, CHCs mostly originate from the same (interior) region of the body (Blomquist and Bagnères 2010), but may then migrate across the cuticular surface via diffusion. Their physical properties, including melting point and viscosity, may influence their diffusion rate across the insect cuticle, possibly generating regional CHC differences only due to differential CHC diffusion (Menzel et al. 2019; Sprenger et al. 2018).

Analyzing body part-specific CHC profiles across further insect species may help determine which mechanism (passive diffusion or active, targeted transport) is more common, and whether the observed patterns are consistent across taxa or vary between species, potentially revealing additional functional roles that CHCs fulfil in different parts of the body. We expected that body parts important for communication, chemical perception and joint lubrication would be enriched in more fluid, lower-melting CHCs. In contrast, regions with higher surface-to-volume ratio (therefore more prone to desiccation) should contain a higher proportion of solid, higher-melting compounds. To this end, we studied the CHC composition of seven different body parts of the desert locust *Schistocerca gregaria* using gas chromatography-mass spectrometry (GC–MS). We analyzed how CHC composition differs between front legs, hind tibia, hind femur, head, abdomen, thorax and antennae, as well as whole-body extracts. In addition, we used solid-phase micro-extraction to compare CHC composition of hind femur, hind tibia, and the hind femuro-tibial joint, in order to determine whether the CHC profile of the joint differs from that of the surrounding body parts, and to identify which CHC classes may be important for lubrication.

## Material and Methods

### Sample Preparation and CHC Extraction

We investigated the desert locust *Schistocerca gregaria* (Orthoptera: Acrididae). It is native to Africa, Arabia, and West Asia, with occasional expansions into South Asia. The CHC profile of *S. gregaria* is predominantly composed of *n*-alkanes and methyl alkanes (Lockey and Oraha 1990), which is in accordance with its occurrence in warm and arid habitats, where effective protection against desiccation is crucial. Subadults of the gregarious form of *S. gregaria* were bought at Futterhaus® Wiesbaden, Germany. *S. gregaria* inhabits regions where daily ground temperatures range approximately from 21 to 55 °C, with air temperatures typically between 22 and 38 °C and relative humidity from 9 to 44%. Nighttime air temperatures range from 16 to 26 °C, with ground temperatures between 14 and 30 °C and relative humidity from 16 to 53% (Maeno et al. 2021). In the laboratory, individuals were maintained at 24 °C and 70% relative humidity conditions and were provided with food in form of grass and oats as well as water ad libitum. Locusts were freeze-killed at −20 °C. Males and females were not distinguished in this study. The body parts of six individuals were separated in antennae, head capsule, thorax, front legs, femur and tibia of the hind legs and the abdomen using a razor blade. For each individual, the left and right antenna were pooled, as well as front legs, hind femora, and hind tibiae, respectively. CHCs of these body parts as well as entire bodies of 5 additional locusts were extracted by immersing them in *n-*hexane for 10 min.

To analyze the CHC composition of *S. gregaria* joints, solid-phase microextraction (SPME) was used. Four individuals were placed into the freezer at −20 °C for one hour, then thawed at room temperature for 30 min. The short freezing period was intended to immobilize, rather than kill, the locusts and to preserve the CHCs in their natural aggregate state. The femuro-tibial joint, tibia or femur of the hindleg of each individual was then stroked with a 100 µm Polydimethylsiloxane coated SPME fiber (Supelco Analytics, Bellefonte, USA) for 10 min to extract the CHCs of these body parts.

### GC–MS Analysis

Hexane extracts of CHCs (2 µl per sample), as well as SPME fibers, were analyzed with gas chromatography-mass spectrometry (GC–MS). We used pure *n*-hexane as a control after every ten runs to ensure that no contamination remained in the column (Fig. S3d). The GC (7890A, Agilent Technologies, Santa Clara, CA, USA) was fitted with a Zebron Inferno ZB5-HT column (Phenomenex Ltd., Aschaffenburg, Germany). We used a single-taper, deactivated splitless liner (Agilent 5181–3316). SPME fibers were left for 10 min in the liner after the temperature programme started. Helium was used as a carrier gas with a flow rate of 1.2 ml/min. The oven temperature started at 60 °C for 2 min, heated up with 60 K/min to 200 °C and then with 4 K/min until 320 °C, when it stayed constant for 10 min. We used a mass selective detector (5975 C, Agilent Technologies) with an ionization voltage of 70 eV. Fragments in the range of 40–550 m/z were detected and used for substance identification. CHCs were identified using diagnostic ions as well as retention indices based on a standard series of n-alkanes (Carlson et al. 1998).

CHCs were quantified based on total ion counts using single ion monitoring. We monitored the ions 55, 57, 69, 71, 82, 83, 85, 96, 97, 99 m*/z*, which are by far the most abundant mass fragments in hydrocarbons and thus allow reliable quantification while at the same time strongly reducing error due to contaminations.

For quantification, all chromatogram peaks were manually integrated using MSD ChemStation (E.02.02.1431, Agilent Technologies). Thus, the total ion counts (based on the single ion monitoring) of each peak were used as a measure of abundance. Trace substances accounting for less than 0.5% of the total profile at maximum across all samples, as well as non-hydrocarbons (which were rare), were deleted from the dataset. Unknown compounds were retained in the dataset unless they could be confidently excluded as non-hydrocarbons. Subsequently, the profiles were standardized to a total of 100%. In the following, we use ‘proportion’ or ‘abundance’ synonymously to refer to the relative proportion (or relative abundance) of a certain CHC class.

### Statistical Analysis

Firstly, we calculated univariate CHC traits from the CHC profiles. These included the proportions of *n*-alkanes, monomethyl, dimethyl and trimethyl alkanes and unsaturated CHCs, as well as the average chain length of *n-*alkanes (weighted by the relative abundance of *n-*alkanes for each chain length). This average chain length was determined for *n-*alkanes, which represent a homologous series, but was not extended to other substance classes, as the position of methyl groups strongly influences melting point (Gibbs & Pomonis 1995), making averages across CHCs with different methyl positions uninformative. These traits were each analysed using a linear mixed-effects model with the trait in question as response variable, *body part* as fixed factor and *individual ID* as random factor. For post hoc tests, we used Tukey tests based on the models (R command *glht*, package *multcomp*).

Secondly, we analysed the CHC composition using a Principal Component Analysis (PCA; R command *prcomp*). For this analysis, data was transformed using Aitchison’s log-ratio transformation to ensure variable independence (Aitchison 1986). Differences between body parts were tested with PERMANOVA using the software PRIMER 6.1.14 & PERMANOVA + 1.0.4 (Primer-E Ltd) (Clarke and Gorley 2015) with 9999 permutations, and *body part* as fixed factor.

For the SPME samples, we analysed the same univariate traits as above. We used linear mixed models with individual ID as random factors but refrained from multivariate methods, due to the low sample sizes. All statistics were done in R 4.5.0 if not mentioned otherwise.

## Results

### Differences in CHC Classes

**Fig. 1:**
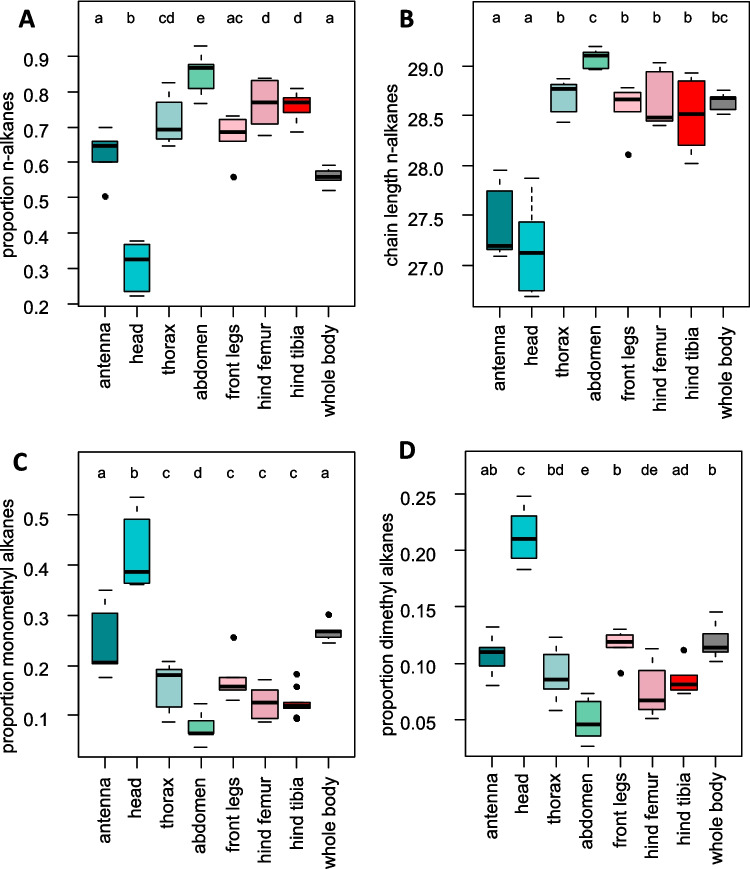
CHC proportions and *n*-alkane chain length per body part. The boxplots show (**A**) proportion of *n-*alkanes, (**B**) average chain length of *n*-alkanes, (**C**) proportion of monomethyl alkanes and (**D**) proportion of dimethyl alkanes for each body part. Different letters indicate significant differences according to post hoc tests of the linear mixed models

We identified a total of 31 CHC peaks across all samples (Table 1). The most abundant compounds were *n*-alkanes, which accounting for 50–90% of the total CHC profile. In addition, we detected several methyl-branched alkanes and a low proportion of unsaturated CHCs. Relative *n*-alkane abundance differed between body parts (LMM: χ^2^_7_ = 535.9, p < 0.0001), being highest in the abdomen ( >70%), followed by thorax, hind tibia and hind femur, and lowest in the head (~ 30%). Furthermore, the average chain length of *n*-alkanes was highest in the abdomen and lowest in antennae and head, but similar across all other body parts (LMM: χ^2^_7_ = 678.45, p < 0.0001) (Fig. 1b).

**Table 1:** Cuticular hydrocarbons of *Schistocerca gregaria*

RT	RI	substance	substance class	CL	diagnostic ions (m/z)	average (%)	number
10.00	21.00	*n-*C21	*n-*alkane	21	296	0.07 ± 0.03	23
11.22	22.00	*n-*C22	*n-*alkane	22	310	0.05 ± 0.02	9
12.94	23.00	*n-*C23	*n-*alkane	23	324	0.49 ± 0.07	65
14.46	24.00	*n-*C24	*n-*alkane	24	338	0.24 ± 0.05	66
16.06	25.00	*n-*C25	*n-*alkane	25	352	3.59 ± 0.49	67
17.66	26.00	*n-*C26	*n-*alkane	26	366	0.52 ± 0.08	63
19.28	27.00	*n-*C27	*n-*alkane	27	380	10.89 ± 0.65	67
20.90	28.00	*n-*C28	*n-*alkane	28	394	1.42 ± 0.08	67
22.50	29.00	*n-*C29	*n-*alkane	29	408	43.38 ± 1.96	67
23.00	29.32	11-MeC29	intMonoMe	29	168/281	0.34 ± 0.02	64
23.64	29.72	3-MeC29	terMonoMe	29	57/393	0.24 ± 0.01	67
24.04	30.00	*n-*C30	*n-*alkane	30	422	1.13 ± 0.06	67
24.56	30.31	unknown CHC	unknown CHC	30	NA	0.21 ± 0.01	66
25.58	31.00	*n-*C31	*n-*alkane	31	436	6.14 ± 0.43	67
25.68	31.05	unknown CHC	unknown CHC	31	NA	0.21 ± 0.06	18
26.06	31.30	11-;9-MeC31	intMonoMe	31	168/308; 140/336	4.53 ± 0.33	67
26.50	31.59	9,13-DiMeC31	DiMe	31	141/351, 211/280	2.25 ± 0.13	67
26.80	31.79	unknown CHC	unknownCHC	31	NA	0.18 ± 0.01	64
27.08	32.00	*n-*C32	*n-*alkane	32	450	0.17 ± 0.01	67
27.54	32.29	11-MeC32	intMonoMe	32	168/322	0.59 ± 0.04	67
27.96	32.58	9,13-DiMeC32	DiMe	32	141/365,211/294	0.38 ± 0.02	66
28.54	33.00	*n-*C33	*n-*alkane	33	464	0.8 ± 0.1	67
29.00	33.29	9-;11-;13-MeC33	intMonoMe	33	141/365;168/337;196/308	9.76 ± 0.83	67
29.42	33.59	9,13-;11,15-DiMeC33	DiMe	33	140/379,239/281	6.9 ± 0.46	67
29.80	33.85	9,13,21-TriMeC33	TriMe	33	141/393,210/323,336/196	0.54 ± 0.04	67
30.40	34.28	9-;11-MeC34	intMonoMe	34	140/379,168/351	0.41 ± 0.03	67
30.80	34.57	9,13-;9,15-;9,17-DiMeC34	DiMe	34	140/393,211/323;140/393,239/295; 140/393,266/267	0.25 ± 0.02	64
31.80	35.29	11-;13-MeC35	intMonoMe	35	168/364;196/336	1.59 ± 0.11	66
31.94	35.39	C35ene	alkene	35	490	0.25 ± 0.04	45
32.02	35.45	C35ene	alkene	35	490	0.08 ± 0.02	15
32.18	35.57	C35ene	alkene	35	490	1.51 ± 0.14	66
32.36	35.70	C35ene	alkene	35	490	0.34 ± 0.03	56
32.48	35.79	7,12,14TriMeC35	TriMe	35	112/448, 196/365,239/323	0.44 ± 0.04	60
34.78	37.50	13-MeC37	intMonoMe	37	196/364	0.14 ± 0.04	37

For each GC–MS peak, the table gives retention time (RT), retention index (RI), substance identification, substance class, chain length (CL), diagnostic ions, the average abundance per sample (mean and standard error, in %), and the number of samples where the compound occurred. Abbreviations for substance class: intMonoMe = internally branched monomethyl alkane, terMonoMe = terminally branched monomethyl alkane, DiMe = dimethyl alkane, TriMe = trimethyl alkane

The proportion of mono- and dimethyl alkanes also differed significantly between body parts (LMMs: monomethyl alkanes: χ^2^_7_ = 412.7, p < 0.0001; dimethyl alkanes: χ^2^_7_ = 338.5, p < 0.0001). Generally, the highest content of methyl alkanes was found in the head, followed by antennae and whole-body extracts, and lowest in the abdomen (Fig. 1b, c). Alkenes and trimethyl alkanes were usually less abundant than 5%, but showed roughly similar differences between body parts as mono- and dimethyl alkanes (LMMs; trimethyl alkanes: χ^2^_7_ = 137.57, p < 0.0001, alkenes: ${\chi }_{7}^{2}$ = 43.8, p < 0.0001) (Fig. S1).

### Differences in Overall CHC Composition

**Fig. 2:**
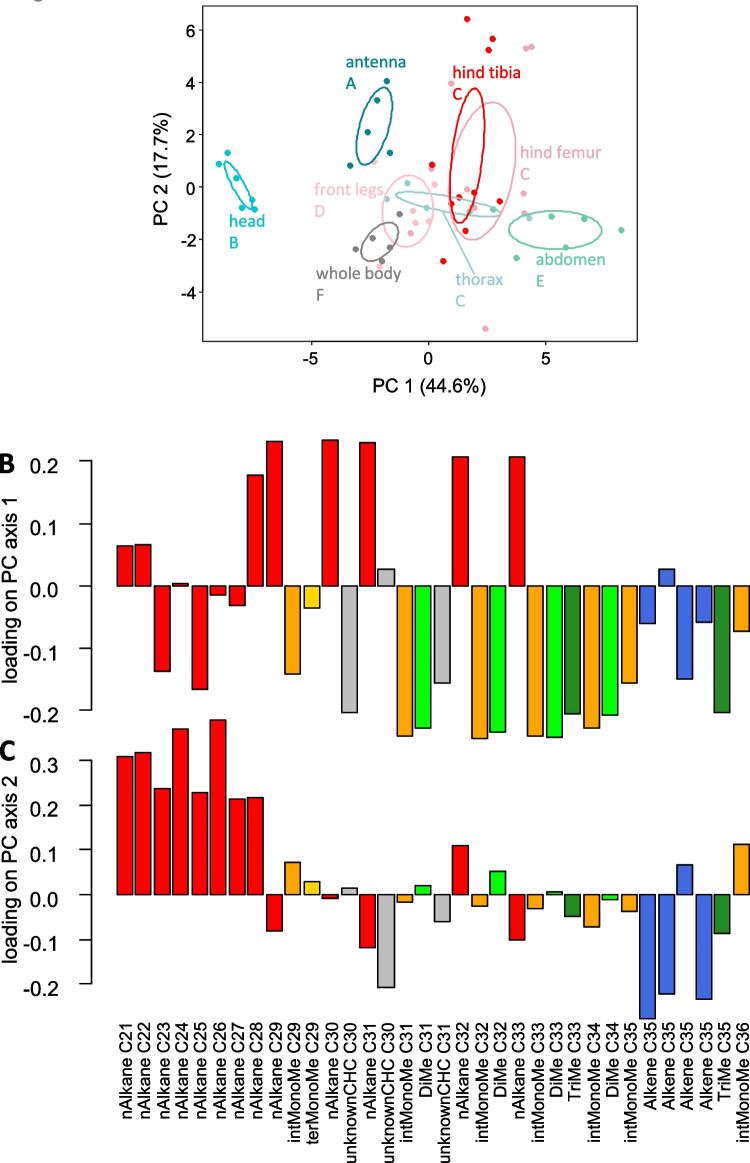
Principal Component Analysis of the CHC composition in different body parts of *Schistocerca gregaria*. Each point represents one sample, color-coded by body part. Ellipses indicate the 95% confidence intervals around the group centroid per body part. The letters denote statistical differences according to PERMANOVA; body parts with same letters are not statistically different. (b) PCA loadings for PC axis 1 and 2 for the main contributing compounds shown per substance (red: *n*-alkanes (nAlkane), orange: internally branched monomethyl alkanes (intMonoMe), yellow: terminally branched monomethyl alkanes (terMonoMe), grey: unidentified CHCs (unkownCHC), light green: dimethyl alkanes (DiMe), dark green: trimethyl alkanes (TriMe), blue: alkenes (Alkene)

Concerning overall CHC composition, body parts differed strongly from each other (PERMANOVA: pseudo-F = 25.1, df = 6, p = 0.0001). In contrast, differences between individuals (pseudo-F = 2.2, df = 11, p = 0.068) or the interaction of body part and individual ID (pseudo-F = 0.50, df = 31, p = 0.97) were not significant. The greatest differences were observed between the antennae and the head capsule, as well as between these two parts and all other body regions. Additionally, the abdomen and whole-body extracts differed from each other and from all other body parts (Fig. 2a). Pairwise, FDR-corrected comparisons from the PERMANOVA indicated that non-significant differences occurred only between thorax, hind femur, and hind tibia.

The first principal component (PC 1) accounted for 44.6% of the variance (eigenvalue: 15.2). Most *n-*alkanes had positive loadings on this axis, whereas all monomethyl and dimethyl alkanes, alkenes, and two shorter-chain *n-*alkanes showed strong negative loadings (Fig. 2b). A similar pattern was observed for the second PC axis (eigenvalue: 6.0; 17.7% explained variance), with most, especially shorter *n-*alkanes showing strong positive loadings, and most long-chain alkenes had negative loadings. In contrast, loadings of most other compounds were comparably small (Fig. 2c). PC1 values were highest for abdomen, hind femur and hind tibia, and lowest for the head, with intermediate values for antennae and whole body (Fig. S2)*.*

### SPME Analyses of Joints

**Fig. 3:**
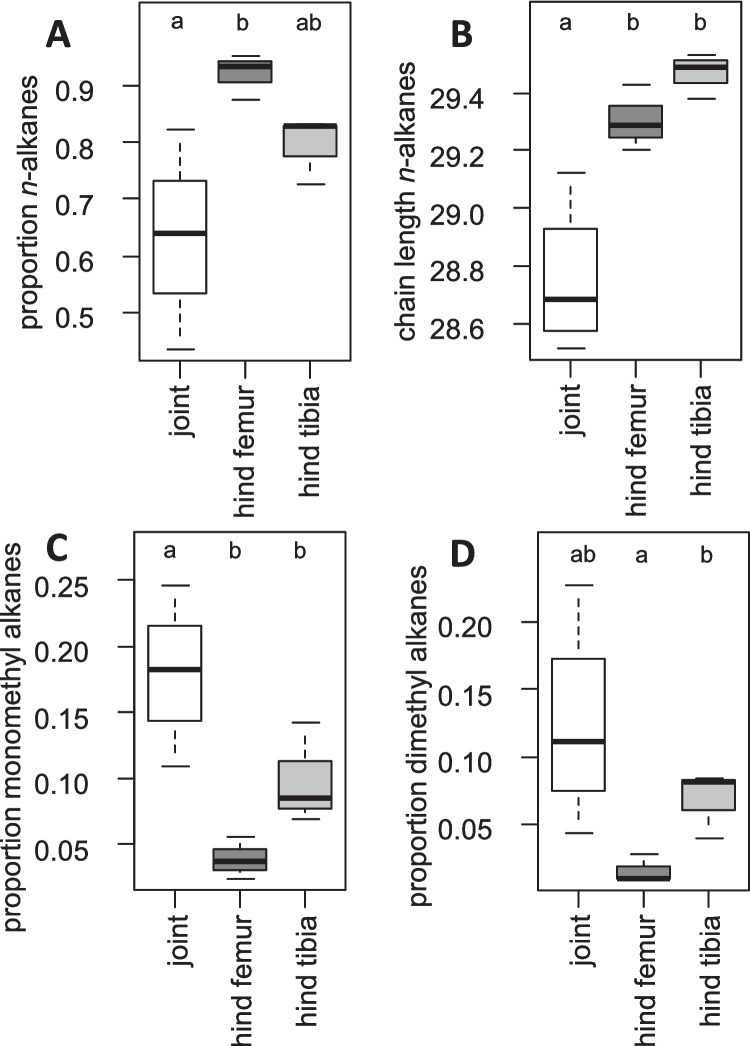
CHC proportions and *n*-alkane chain length in and around joints, as evidenced by SPME extracts. The boxplots show (**A**) proportion of *n-*alkanes, (**B**) average chain length of *n*-alkanes, (**C**) proportion of monomethyl alkanes and (**D**) proportion of dimethyl alkanes for femoro-tibial joint, hind femur and hind tibia. Different letters indicate significant differences according to post hoc tests of the linear mixed models

Joints, hind femur and hind tibia differed little in substance class composition. Differences were significant for *n*-alkane content (LMM: χ^2^_2_ = 11.9, p = 0.0026) and average *n*-alkane length (LMM: χ^2^_2_ = 28.7, p < 0.0001), as well as for monomethyl alkane content (LMM: χ^2^_2_ = 18.8, p < 0.0001) and for dimethyl alkanes (LMM: χ^2^_2_ = 14.6, p = 0.00067). Compared to the femur, joints had less and shorter *n*-alkanes, and more monomethyl alkanes (Fig. 3). Joints differed in a similar way from the tibia, only that *n*-alkane proportion was not significantly different.

## Discussion

We found significant CHC variation between body parts of the desert locust *Schistocerca gregaria*. Notably, antennae and head were most different from all other body parts. They had the lowest relative *n-*alkane abundance and the shortest *n-*alkane chain length. Both findings suggest that the CHCs of these body parts have low melting points. This coincides with Wang et al. (2016b), where antennae and head CHCs (especially of the mouth parts) also had the lowest melting points compared to other body parts. That antennal CHCs are most different from other body parts has also been found for *Iridomyrmex* ants (Wang et al. 2016a). This aberrant CHC composition of antennae appears plausible given that antennae not only serve as primary sensory organs for detecting chemical and tactile cues, but also carry recognition cues themselves. Ants often antennate the opponent's antennae first but have difficulties recognising non-nestmates that lack antennae (Wang et al. 2016a). Recognition cues should be easy to perceive for another insect’s antennae and hence should be as fluid and transmittable as possible (Menzel et al. 2019). Less viscous CHCs have by definition higher diffusion rates (Einstein 1905) and thus should diffuse more easily across the body surface and also diffuse faster towards another individual’s odorant receptors during contact chemoreception. Moreover, less viscous CHCs should exhibit higher vapor pressures compared to more viscous CHCs, i.e. have more molecules entering the gas phase (Othmer and Conwell 1945). This would again facilitate olfactory reception by another individual. Although their volatility is very low, insect CHCs were shown to be volatile enough to be detected by other insects in the gas phase, without physical contact (Brand-staetter et al. 2008). Thus, low-viscosity compounds should be easier to perceive by another individual’s olfactory receptors, thereby enabling effective chemical communication. This may explain why antennal profiles were enriched in lower-melting compounds. Interestingly, CHCs accumulate rapidly on the antennae if insects are prevented from grooming them (Böröczky et al. 2013). This has been observed in various insect species, and additionally suggests that more fluid CHCs can accumulate in the antennae due to diffusion, while more solid CHCs diffuse less far and remain closer to their site of origin in the fat body.

In contrast, it remains uncertain why the head contains more fluid CHCs than other body parts. We suggest that this could be explained by the presence of more fluid CHCs on the mouthparts, such as the maxillae and mandibles, which were included in the head extract in this study. Results from Wang et al. 2016b showed that CHCs of the mouthparts of *Drosophila melanogaster* already melted at 18 °C, like CHCs of the antennae. This may facilitate chemical perception in the maxillary palps which are an important gustatory organ for food detection and assessment (Sevarika and Romani 2025). In this study, the inclusion of the mouthparts in the head extracts could therefore substantially increase the apparent relative abundance of alkenes and other fluid CHCs.

The higher proportion of fluid CHCs on head and antennae is consistent with the idea that high-melting *n*-alkanes diffuse less rapidly across the insect's body compared to lower-melting CHC classes. Fat bodies, where the CHCs are produced (Blomquist 2010), are mostly located in the abdomen. Thus, the longer chain lengths of *n*-alkanes observed on the abdomen further support the idea of differential diffusion rates, as increased chain length is known to increase melting points (Gibbs and Pomonis 1995). Consistent with this, body parts more distant from the abdomen, such as the head and antennae showed a higher proportion of shorter, and thus more fluid, CHCs. In summary, our findings are consistent with the hypothesis that differential diffusion rates contribute to variation in CHC composition across body regions.

The CHC composition of the remaining body parts was relatively similar, which is largely consistent with the findings of Wang et al. (2016b). Note however that their study revealed differences between the dorsal side, characterized by higher-melting substances, and the ventral side, which had lower-melting compounds.

The dominant CHC class in *S. gregaria* are *n*-alkanes which represented up to 90% of the total CHC amount. Compared to other CHC classes, *n*-alkanes exhibit the highest melting temperatures, and are usually solid at room temperature. As solid CHCs offer superior protection against desiccation compared to liquid phases, *n*-alkanes serve as an effective barrier against water evaporation. This fits the habitat of *S. gregaria*, which inhabits semi-arid to arid deserts (Guan et al. 2021). *Schistocerca* habitats have been shown to exhibit ground temperatures up to 55 °C and air temperatures (in 2 m height) up to 38 °C (Maeno et al. 2021); thus, the high proportion of late-melting *n-*alkanes seems adaptive. It should be noted that humidity and temperature are known to influence the composition of the CHC layer (Sprenger et al. 2018; Hadley 1977; Gibbs & Mousseau 1994). Therefore, the quantitative composition of CHC profiles of locusts maintained under laboratory conditions may differ from those of locusts in their natural environment, albeit the qualitative composition is usually species-specific and hardly influenced by environmental conditions (Sprenger & Menzel 2020). Elevated *n*-alkane levels have also been observed in other desert-dwelling insects, such as the locust *Locusta migratoria* (Lockey and Oraha 1990; Yang et al. 2020) and the scorpion *Buthus occitanus* (Gefen et al. 2015). Together, this corroborates the notion that *n*-alkanes are necessary for desert arthropods to minimize water loss.

### CHC Profile of Joints

CHCs have been hypothesized to serve as lubricants in joints (Cooper et al. 2009). Indeed, the femoro-tibial joint of *S. gregaria* exhibited the lowest proportion of *n*-alkanes and the highest proportion of alkenes compared to femur and tibia. Statistical significance for CHC content was found only between the joint and femur for *n*-alkanes, whereas chain length differences were significant, with both the femur and tibia differing from the joint. Relative alkene abundances tended to be higher in the joint, but this was not statistically supported, possibly due to the limited sample size. This occurrence of earlier-melting CHCs in the joint may reflect their function as a lubricant in this region. However, joints may also contain other lubricating substances, which are chemically distinct from CHCs (Nadein et al. 2021). Hence, it remains unclear whether CHCs are the primary lubricant in insect joints, although our results are consistent with this hypothesis.

## Conclusion

In summary, we showed that CHC composition varies across body parts. Antenna and head (including mouth parts) exhibited the most aberrant CHC composition, characterized by the highest proportion of more fluid CHCs. This corresponds with previous studies (Böröczky et al. 2013; Wang et al. 2016b) and supports the functional role of the antennae as sensory organs involved in chemical communication. In contrast, the other body parts displayed a largely homogenous CHC composition. Analysis of the femoro-tibial joint revealed trends toward increased relative abundance of more fluid CHCs, suggesting a potential role of CHCs as lubricants.

Body part-specific CHC profiles may hence be explained by local functional requirements (chemoreception, lubrication, protection from water loss). Future studies could address whether all differences are adaptive or may also be a by-product of differences in diffusion rates. Furthermore, more detailed studies on CHC differentiation across finer body parts (e.g. tarsi, mouth parts) may reveal the role of intra-individual CHC variation for its multifunctionality.

## Supplementary Information

Below is the link to the electronic supplementary material.

Supplementary file1 (PDF 378 KB)

Supplementary file2 (XLSX 36 KB)

## Data Availability

All raw data will be made available in the supplement of this manuscript upon acceptance of the manuscript.
