# Supplementary material for: Cuticular Hydrocarbon Differentiation Between Body Parts of Schistocerca gregaria Locusts
Source: J Chem Ecol. 2026 Feb 12;52(1):16. doi: 10.1007/s10886-025-01687-y (PMC12901264; doi:10.1007/s10886-025-01687-y)
Supplement: Supplementary file 1 — Supplementary file1 (PDF 378 KB) [file 10886_2025_1687_MOESM1_ESM.pdf]

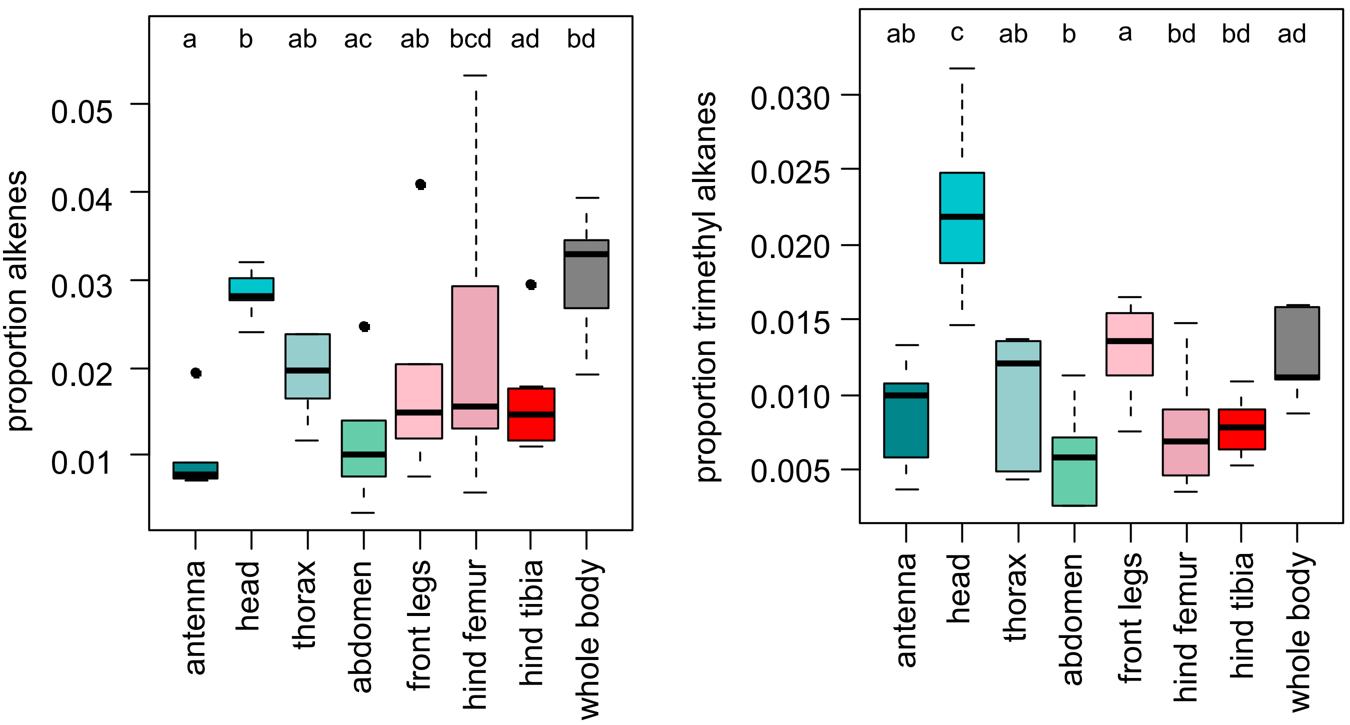

Fig. S1 Proportions of alkenes and trimethyl alkanes for each body part. Plots with same letters are not significantly different according to post hoc tests of the linear mixed-effects models.

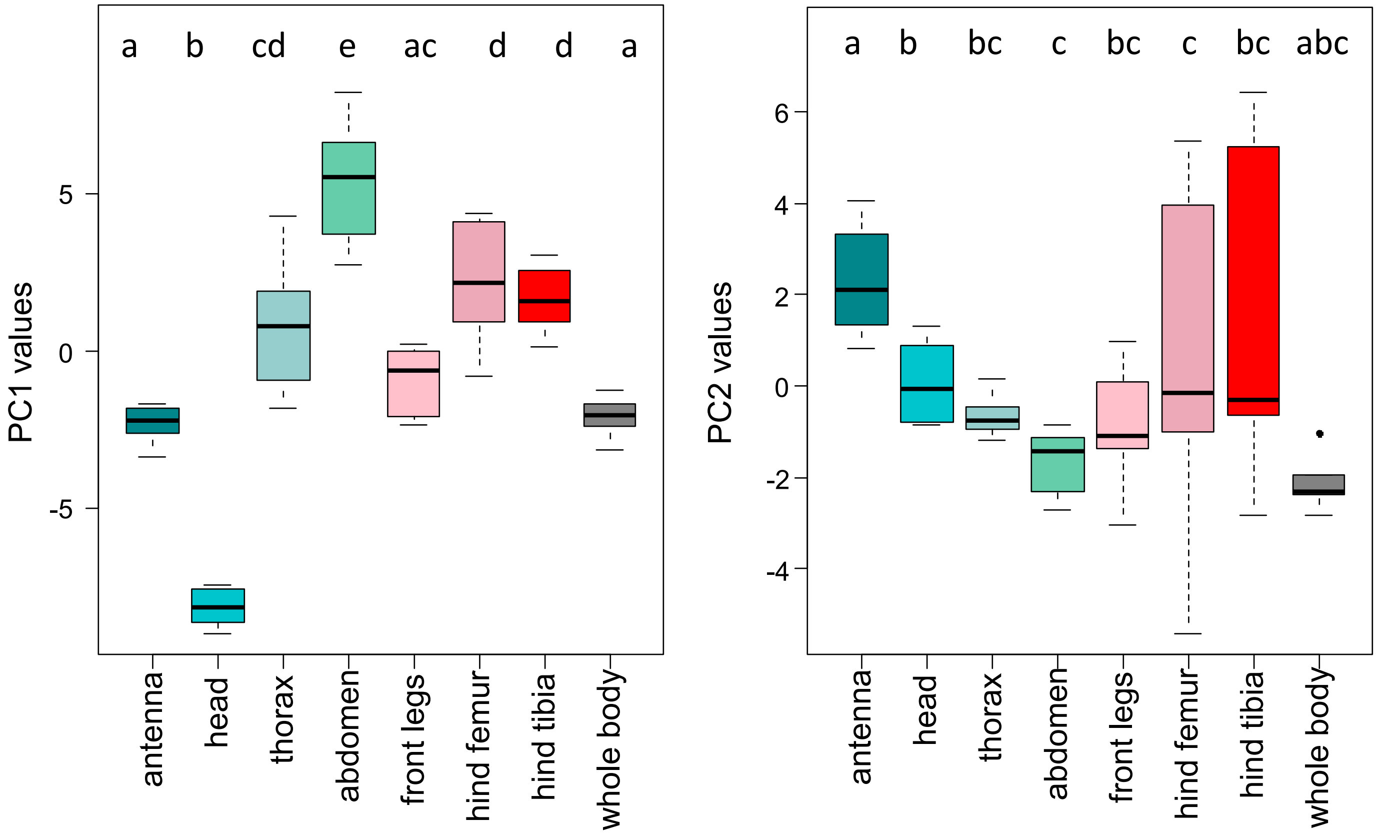

Fig. S2 PCA values of PC1 and PC2 for each body part. Higher positive values indicate samples with higher contributions of variables loading positively on the PC axis, while negative values indicate samples with higher contributions of variables loading negatively on PC axis. Plots with same letters are not significantly different according to post hoc tests of the linear mixed-effects models.

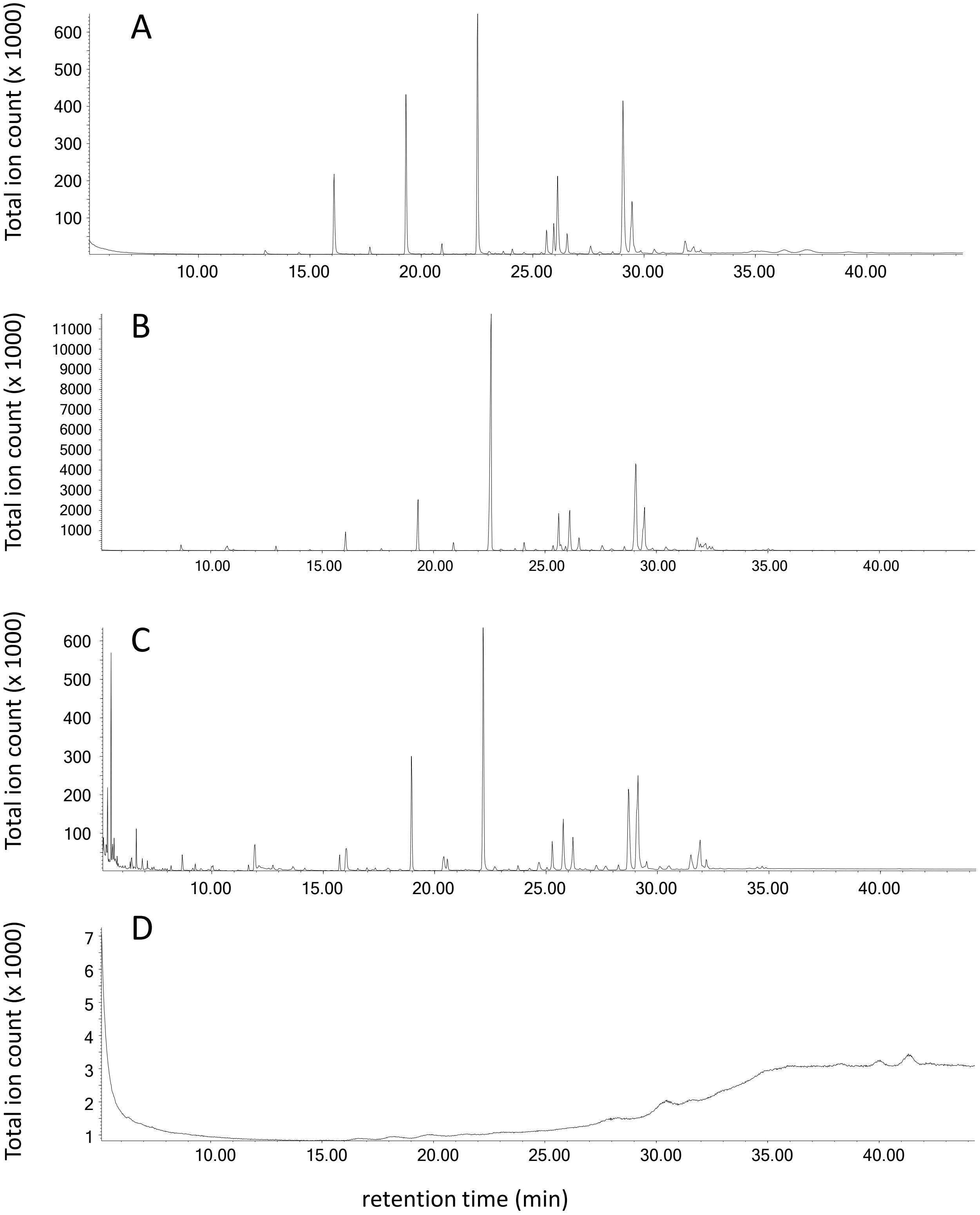

Figure S3 Gas chromatograms of three *S. gregaria* body parts and a hexane control. (A) antennae, (B) whole body, (C) Joint (SPME), (D) hexane control run. Note the different scales of the Y axis.
